# Metamodels for Transdisciplinary Analysis of Wildlife Population Dynamics

**DOI:** 10.1371/journal.pone.0084211

**Published:** 2013-12-13

**Authors:** Robert C. Lacy, Philip S. Miller, Philip J. Nyhus, J. P. Pollak, Becky E. Raboy, Sara L. Zeigler

**Affiliations:** 1 Chicago Zoological Society, Brookfield, Illinois, United States of America; 2 IUCN SSC Conservation Breeding Specialist Group, Apple Valley, Minnesota, United States of America; 3 Colby College, Waterville, Maine, United States of America; 4 Information Science, Cornell University, Ithaca, New York, United States of America; 5 Department of Ecology and Evolutionary Biology, University of Toronto, Toronto, Ontario, Canada; 6 Department of Biological Sciences, Virginia Tech, Blacksburg, Virginia, United States of America; SUNY College of Environmental Science and Forestry, United States of America

## Abstract

Wildlife population models have been criticized for their narrow disciplinary perspective when analyzing complexity in coupled biological – physical – human systems. We describe a “metamodel” approach to species risk assessment when diverse threats act at different spatiotemporal scales, interact in non-linear ways, and are addressed by distinct disciplines. A metamodel links discrete, individual models that depict components of a complex system, governing the flow of information among models and the sequence of simulated events. Each model simulates processes specific to its disciplinary realm while being informed of changes in other metamodel components by accessing common descriptors of the system, populations, and individuals. Interactions among models are revealed as emergent properties of the system. We introduce a new metamodel platform, both to further explain key elements of the metamodel approach and as an example that we hope will facilitate the development of other platforms for implementing metamodels in population biology, species risk assessments, and conservation planning. We present two examples – one exploring the interactions of dispersal in metapopulations and the spread of infectious disease, the other examining predator-prey dynamics – to illustrate how metamodels can reveal complex processes and unexpected patterns when population dynamics are linked to additional extrinsic factors. Metamodels provide a flexible, extensible method for expanding population viability analyses beyond models of isolated population demographics into more complete representations of the external and intrinsic threats that must be understood and managed for species conservation.

## Introduction

### The Evolution of PVA Modeling

The dynamics of wildlife populations are highly complex due to the diversity of processes that drive ecological systems, branching chains of causal effects, feedbacks, coupling between human and natural systems, and other often non-linear interactions [1]. Ecological models aim to represent natural systems in a manner that is complete enough to forecast system dynamics with accuracy yet simple enough to reflect limitations of data and knowledge [2,3]. Although simplified models facilitate a general understanding of ecological systems, such models may not incorporate the true dynamics in sufficient detail for conservation management[4,5] because they ignore sources of uncertainty or stochasticity [6,7] or other critical factors (e.g., species-specific life history attributes and dispersal behaviors [8–10]; social behaviors [11–13]; genetics [14]). 

Many recent species extinctions have been caused by multiple, interacting, human-induced stresses [15] (e.g., pathogens, invasive species, collapse of inter-connected communities, and global change). However, such stresses are rarely incorporated fully into conservation planning because of a lack of understanding of how threats act on a species [16] and because populations are often threatened by multiple stressors interacting synergistically [17]. The development of analytical tools for population viability analysis (PVA) has facilitated the consideration of both deterministic and stochastic threats [18,19]. However, the restricted array of threats typically included in PVA models (e.g., demographic and environmental stochasticity, genetic decay, and habitat loss) limit the ability of these models to describe even some primary biological, physical, and human forces acting on wildlife populations [20–22]. For example, most PVA models target a single species [23] under implicit assumptions that the species does not interact with other species or that the interacting species are constant in number or do not respond through dynamic feedback processes. Thus, processes such as coupled predator-prey systems, competition-structured communities, and infectious disease are rarely included in PVA models. Moreover, most PVA models use data from field studies focused on single populations over short time scales to provide estimates of demographic rates with the assumption that past threats are adequate predictors of the future. Given the rate at which the global environment and local human impacts are changing [24], population models must consider the changing forces driving population and system dynamics [1,25]. 

The need to expand PVA methods has been previously recognized. Authors have called for the inclusion of more threats in viability assessments [9,21], more explicit handling of the sources of uncertainty [26,27], improvements to collaborative processes through which PVAs are applied to conservation [28,29], incorporation of multiple disciplinary perspectives [20], and the integration of PVA with other tools for more complete strategic planning [30,31]. However, methods for extending PVA into a broader transdisciplinary approach and the tools needed to implement such an approach have not been readily available. 

### Metamodels: A New Approach for Extending PVA

Previously, we conceptualized what we term “metamodels” to facilitate more holistic analyses of the diverse threats acting on wildlife populations [32,33]. The metamodel approach links discipline-specific models representing components of an overall system to reveal emergent properties of multi-dimensional interactions. In this approach, a central facilitator program controls the sharing of information between models (i.e., the outputs of one model can be inputs for another), manages the sequence of events in the overall simulation, translates variables into a common language for all linked models, and ultimately combines outputs into a meaningful representation of results. This approach is transdisciplinary in that the interactive data flow allows projections of non-linear feedbacks among processes that are analyzed by diverse disciplinary methods. Thus, it provides more than a multi-disciplinary summed effect of independent analyses or an inter-disciplinary analysis of the connections between processes. It combines the methods and strengths of each discipline into a more encompassing analysis of main effects, interactions among effects, and emergent higher level dynamics. It provides a methodology for what Holling [34,35] described as the “science of the integration of parts”.

**Figure 1:**
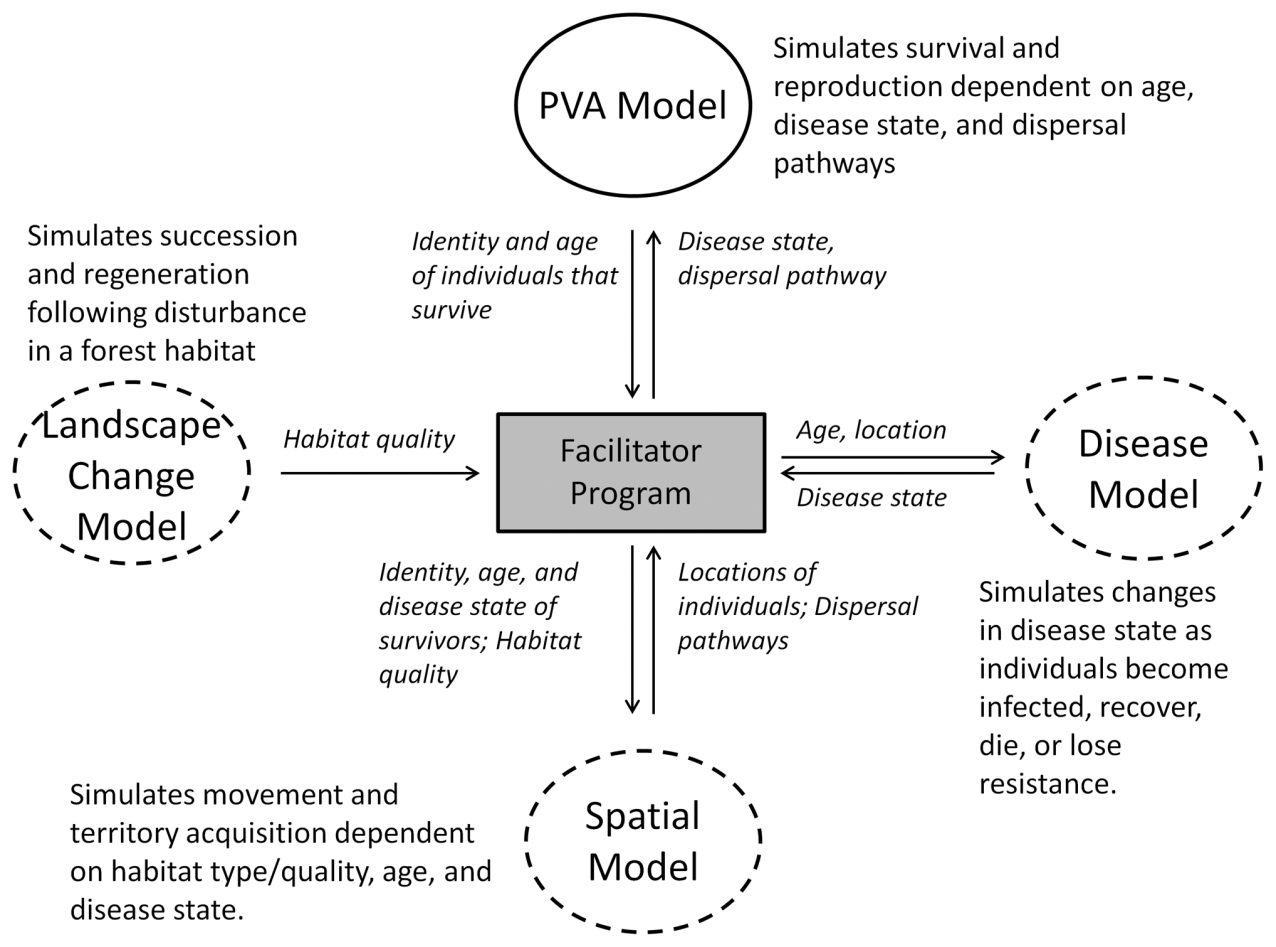
Metamodel that integrates demography, landscape change, dispersal, and disease status. A PVA program acts as the system model (solid outline) to simulate individual survival and reproduction based on individual and population state variables (shown in italics) passed from other models. Modifier models (dashed outlines) simulate habitat dynamics, individual movements, and individual transitions in disease status. A central facilitator program passes state variables between the system and modifier models at appropriate time steps. The ultimate results are measures of population dynamics and extinction risk for a species impacted by habitat change and disease.

The metamodel approach follows one of Nicholson et al.’s [36] heuristics for interdisciplinary modeling – using a suite of sub-models rather than one all-purpose model. This approach helps to overcome challenges inherent to the use of independent models that lack dynamic interactions or, alternatively, the development of a massively complex model. Independent models can provide insights into each of the processes impacting a system in isolation. However, that approach might not identify the relative importance of those processes nor elucidate if synergisms in those processes have cumulative impacts that differ from the impacts predicted from individual processes. Without an integrated analysis, uncoupled assessments can result in contradictory conclusions regarding likely trajectories and effective management actions. In contrast, a metamodel yields a single outcome resulting from multiple forces interacting through dynamic feedbacks, ultimately balancing any contradictory results generated by uncoupled models. For example, a metamodel that combines a wildlife population model, a habitat change model, and an epidemiological model of infectious disease (Figure 1) can be used to examine how changes in habitat connectivity can alter dispersal, thereby altering disease spread and source-sink dynamics within a metapopulation. It would be difficult to assess the dynamics of a system like this from any one perspective because the population model in isolation would likely predict that habitat fragmentation would destabilize the system [37–39], whereas the disease model would predict that fragmentation would protect local populations from infection [40,41]. The balance of these trade-offs in even this simple case would be hard to predict without a metamodel approach. 

To accommodate interactions, researchers can develop “megamodels” to represent multiple system components within a single model (e.g., FLORES [42] and HexSim [43]). Although megamodels have utility (e.g., global climate models [44]; general ecosystem models [45]), many researchers lack the resources to develop and operate the most expansive models. Conversely, the metamodel approach enables users to examine results of individual component models where there is a high degree of disciplinary confidence, explore interactions among modeled processes, and test alternative configurations to understand how component models and their interactions contribute to overall dynamics. This type of sensitivity analysis can be difficult in a megamodel. 

A lack of common language—or data “translators” that can manage the flow of different data formats at varying spatiotemporal scales—typically prevents the integration of existing disciplinary models into a single transdisciplinary tool. A metamodel can act as this translator, encourage broader analysis by opening each discipline’s models to input from others, and stimulate the development of new models that describe additional aspects of a species and its interaction with its environment.

Linked models can act on different spatial and temporal scales, thereby facilitating analysis of effects that cross scales. For example, a climate model might be used to predict shifts in land cover, informing a landscape model that projects changes in habitat configuration [46] and metapopulation structure [47], which could then inform an agent-based model of animal movements. The dispersal patterns might determine mating patterns in a demographic model, which feeds back up through a genetic model to generate the genetic structure of the population, thereby evaluating the adaptability of the population to climatic changes. Crossing temporal scales, a landscape model might assess changes at a decadal interval, a genetic model might work on a generational time step, a demographic model might use an annual cycle, and a disease model might require daily assessment of disease state transitions. A metamodel can invoke models acting on shorter timescales multiple times within each time step used by the longer-scale models. Linking across spatial scales, metamodels provide a mechanism for down-scaling regional or local patterns of broader global processes to the impacts that subsequently occur on individual populations and their habitats. At the same time, by building models of metapopulation complexes and multi-species interactions, the metamodel approach allows a user to up-scale from population viability models to ecological community and landscape level assessments of cumulative impacts over wider geographic and biological scales.

We describe below a new tool for implementing the metamodel approach. We then present two case studies that demonstrate how metamodels can reveal outcomes from interacting systems that would otherwise be difficult to examine. Our goal is that these descriptions and examples will encourage other researchers to exploit the metamodel approach and to develop additional tools for implementing metamodels.

## Methods

### A New Tool for Metamodel Analyses

We developed a generic platform, *MetaModel Manager* [48], to implement the metamodel approach. The software facilitates a link between simulations of one or more populations with any number of additional “modifier” models that create, use, and modify characteristics of individuals, populations, or environments. Model linkages that are currently being developed and tested by the authors and our collaborators include models of multi-species interactions, wildlife harvest, infectious disease, vegetation change, landscape change, genetics, and animal movements. Details on the use of *MetaModel Manager* are provided in the software manual [49]. 

**Figure 2:**
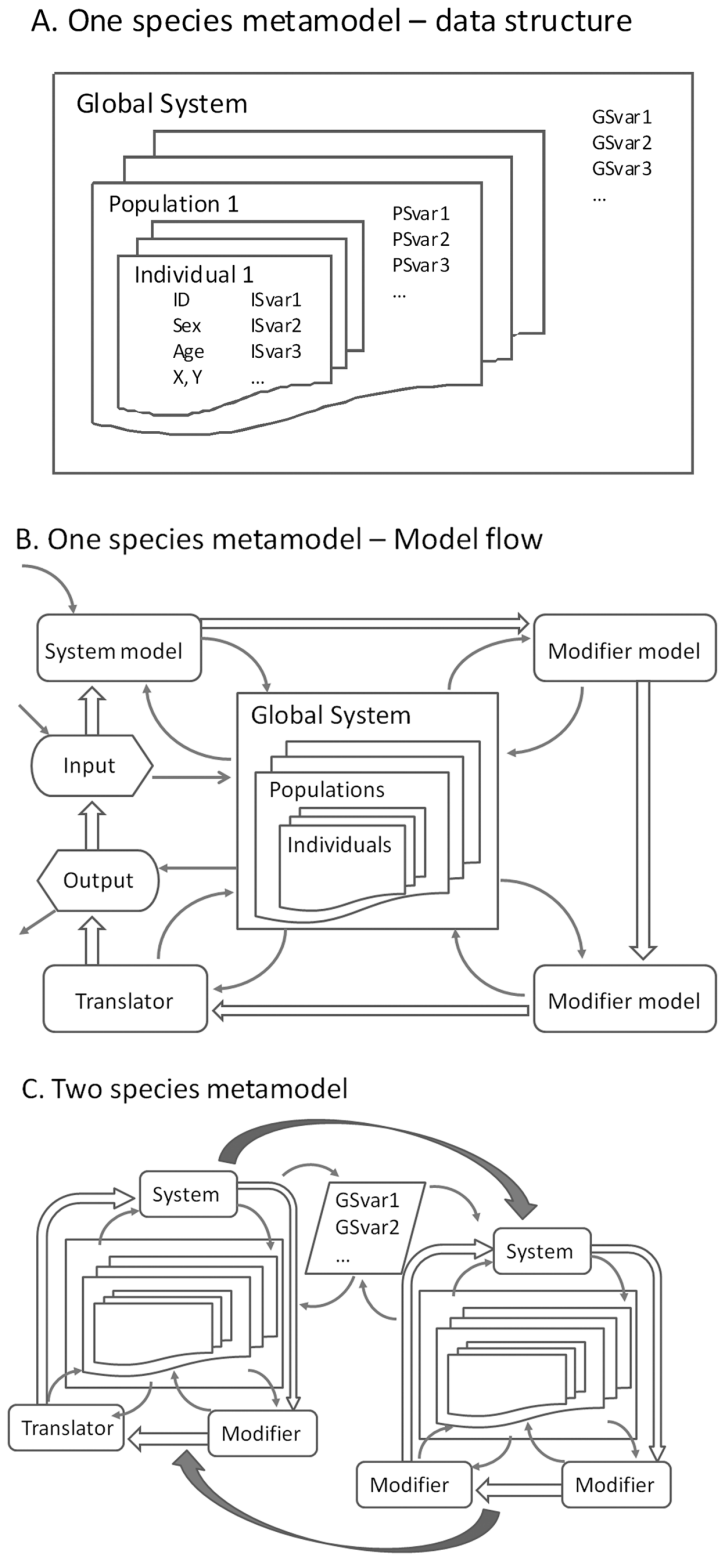
Examples of data structure and program flow implemented by *MetaModel* *Manager*. (A) Nested data representing a species in a metamodel. Global state variables (GSvar), population state variables (PSvar), and individual state variables (ISvar) are descriptors of the overall system, each population, and each individual, respectively. (B) Flow of control among component models. Curved arrows represent access to and modification of data. Block arrows represent control passed among models. (C) A two-species metamodel, with one modifier and one translator model acting on one species and two modifier models acting on the second species. Control alternates between the species, as illustrated by solid block arrows. Each system, modifier, and translator model has access to change any property of its populations and individuals as well as any shared global state variables.

*MetaModel Manager* provides a means of linking several nested levels of data and several types of models (Figure 2). “System models”, at least one of which is required for *MetaModel Manager* to run, initially define the population(s) and its individuals. The data managed by *MetaModel Manager* include, at a minimum, the population’s size and age-sex structure. In individual-based simulations, each individual must be defined with an identifier, age, sex, and living status. The system model can add variables that describe additional characteristics of populations (population state variables) or individuals (individual state variables). The overall system can also have associated global state variables that describe properties that apply to all populations in the focal system. In a PVA context, the system model would normally simulate demographic events (birth, aging, and death). The system model can be as simple as a projection of exponential population growth or as complex as an individual-based simulation. 

Optional “modifier models” add individual, population, or global state variables to the metamodel and apply algorithms that describe transitions in those variables. For example, modifier models might be used to implement transitions in each individual’s social status or spatial location; population variables such as current habitat quality or prey abundance; or global variables such as climate or human population abundance. The separation of these sub-model algorithms into modifier models as opposed to their inclusion within the system model is optional (as all sub-models could be included in more expansive system models). However, this separation is consistent with the metamodel philosophy that promotes the compartmentalization of processes for independent, single-discipline model development, easier model validation, and clearer testing of the role of each model in overall metamodel dynamics. It also encourages the addition of new components as needed without requiring alterations to the computer codes of other models. 

Transformations, which change any individual, population, or global state variable outside of the system and modifier models, can be applied via the evaluation of equations or macros that contain sequential equations in which the other variables are operands. These transformations can function as: (1) translators among the data formats used by different system or modifier models (2), simple modifier models that apply transitions to an individual’s characteristics during a simulation, or (3) a means of acquiring summary statistics from the metamodel. 

Input files read by *MetaModel Manager* at each simulated time step can provide time series of global or population state variables that drive processes in the metamodel, allowing for links between models that do not require interlaced feedback. For example, climate or landscape models could be used to provide projections of change in temperature or habitat, respectively, to a population model for assessing extinction risk in changing environments. Hunter et al. [50] used projections of Arctic sea ice from general circulation models to drive stochastic matrix models of polar bear demography. Similarly, if the effect of one species on another is largely one-directional (e.g., a tree species provides an essential resource to a bird species but is not affected by the presence of the bird), then input files can be a means of providing abundance projections for the resource species to the model of the dependent species.

Finally, *MetaModel Manager* provides the user with the option to pause a simulation and manually modify individual, population, or global variables, thereby allowing the user to become yet another “model” involved in a simulation. In this way, a metamodel can include queries of experts for management decisions based on the status of the population, or the metamodel could be used as a teaching tool to allow users to test the effectiveness of their responses. 

Some key models have already been integrated as options within *MetaModel Manager*, and additional models can be added by users via the program’s interface. These models include the PVA platform *Vortex* (system model) [51,52]; *Outbreak*, which simulates infectious disease epidemiology (as a modifier or system model) [53]; and *Spatial*, which simulates animal movement on landscapes (modifier model) [54]. Metamodels can also allow linkages between individual-based models (e.g., *Vortex*) and population-based models (e.g., *RAMAS Metapop* [55]) to extend local analyses to the landscape scale or to understand interactions between species best modeled at the level of a single population and species best modeled at a metapopulation scale. 

#### Technical specifications

*MetaModel Manager* was developed in the C# language using Microsoft Visual Studio 2010 (Microsoft Corp., Redmond, Washington), with some graphical tools obtained from ComponentOne Studio for WinForms (Grape City Inc., Pittsburgh, Pennsylvania). The program was compiled to run on any version of the MS Windows operating system. An installation package that includes the executable program, a preliminary manual, the *Vortex* PVA program, the *Outbreak* epidemiological model, a macro editor (*MMMacro*), and sample projects is available for free download at www.vortex10.org/MMMInstallation.msi. Further information about these programs is available at www.vortex10.org/MeMoMa.aspx. A folder with the source code for *MetaModel* Manager, *MMMacro*, and *Outbreak* is available at www.vortex10.org/MMM.zip and is archived on the SourceForge (Dice Holdings, Inc.) public repository (at www.sourceforge.net/projects/metamodelmanager/ ). As an alternative to linking models into a metamodel via shared C# classes, data can be passed between *MetaModel Manager* and external models at each step of the overall simulation via text files. This flexibility removes the necessity for linked models to access .NET classes and permits the external models to be programmed in any language that can be compiled into Dynamic-Link Libraries of functions for use by programs running on a Windows-based system. Information about each external model required by *MetaModel Manager* is provided via a simple XML specification file, which can be built from a utility within the *MetaModel Manager* interface.

### Case Study I: A Two-Component Metamodel

To illustrate how a metamodel has the ability to reveal ecological dynamics that would be difficult to predict from unlinked models, we present a hypothetical case in which an infectious disease was introduced to a metapopulation consisting of 10 subpopulations with total abundance N = 250. In the metamodel, population viability was predicted within the PVA software *Vortex* (version 10.0), disease processes were simulated within the epidemiological model *Outbreak* (version 2.0), and the model processes were linked via *MetaModel Manager*. We applied demographic rates that would result in a projected long-term average (deterministic) exponential growth rate of r = 0.042 and a mean generation time of 5.2 years. We added annual fluctuations in demographic rates, specified that this temporal variation was independent across subpopulations, and imposed moderate inbreeding depression in juvenile survival (3.0 “lethal equivalents” [56]). The model was initialized with 10 subpopulations, each with an initial abundance of 25 individuals distributed according to a stable age distribution and a carrying capacity of 50 individuals. We tested a range of dispersal rates (0%, 1%, 2%, 4%, and 10%), which describe the probability that an individual will disperse in any given year to another subpopulation. 

The disease model was initiated with an infected individual in 5 of the 10 subpopulations. Transmission occurred through contact such that an infectious individual had a 10% probability per day of encountering any given individual within its sub-population. We assumed that 10% of those encounters resulted in disease transmission. Occasional infection from an environmental source (e.g., another species) was modeled to occur with a daily contact probability of 0.00274 (once per year per individual) and a transmission rate of 0.0008 per contact (such that one individual in the metapopulation would be infected from an outside source approximately every 5 years when the population size was near its initial N = 250). Infected individuals became infectious after an incubation period of 25 days, remained infectious for an additional 25 days, and then either recovered with 95% probability or died. Recovered individuals retained immunity for 25 days. Each case was simulated for 50 years and repeated with 1000 iterations. 

We simulated population viability under three model structures. In the first, we modeled the metapopulation in *Vortex* alone, with no consideration of disease. In the second scenario, we used a metamodel linking *Vortex* and *Outbreak* but minimized stochasticity in *Vortex* to represent a mostly disease-driven model; we did not include variation in reproductive success among breeding females, annual environmental variation in mortality rates, or inbreeding depression. However, random variation in sex ratio and demographic stochasticity (due to binomial sampling from constant reproductive and survival rates) are intrinsic to individual-based models and could not be disabled. In the final scenario, we simulated metapopulation viability using a full *Vortex – Outbreak* metamodel with stochastic variation, including annual variation in demographic rates and inbreeding depression. The *Vortex* input file that includes all demographic rates, the *Outbreak* disease specification file, and *MetaModel Manager* control files used in this test are available at www.vortex10.org/MetapopDzDemo.zip and in the *MetaModelManager* project on the SourceForge repository.

### Case Study II: A Two-Species PVA

We developed a simple predator-prey metamodel to illustrate how metamodels can be used to extend PVA to encompass dynamic species interactions. In one *Vortex* project, a predator population was modeled with demographic rates typical of a large cat (e.g., puma), resulting in an intrinsic rate of population growth of r = 0.123 when there is no prey limitation. We added annual fluctuations in demographic rates and imposed moderately strong inbreeding depression (6.0 lethal equivalents). We also added a catastrophe (representing occasional disease epidemics) that occurred with 10% probability each year, killing 10% of the animals and reducing breeding by 10% in catastrophe years. We set initial predator population size at N(predator) = 50 with a carrying capacity of K(predator) = 250. We modeled the dynamics of a second species (the prey species; e.g., a deer) in a separate *Vortex* model, with demographic rates that result in an intrinsic rate of population growth of r = 0.142 in the absence of predation. We added annual fluctuations in demographic rates of the same magnitude as for the predator. We set the initial prey population at N(prey) = 10000 with K(prey) = 25000. 

We specified the linkage between the predator and prey with a logistic function that described the increasing number of the prey killed (up to 25) per year by a predator, dependent on the current density of this prey species, and a second logistic function that described the reproductive rate of the predator as dependent on the number of prey consumed. This logistic function for reproduction specified that a predator was physically able to reproduce after consuming a minimum of 5 prey per year but that the predator did not reach full reproductive capability until at least 25 prey were consumed in a year. It was presumed that a predator could survive on alternate prey species (not modeled and therefore assumed not to be significantly impacted by the predator) but availability of the preferred prey was required to raise litters. The mortality of the prey species was defined as the baseline mortality plus the probability of being killed by a predator. The input files used in this case are available at www.vortex10.org/PredatorPreyDemo.zip and on the SourceForge repository.

## Results

### Case Study I

#### Impacts of stochastic processes on dynamics of fragmented metapopulations

**Figure 3:**
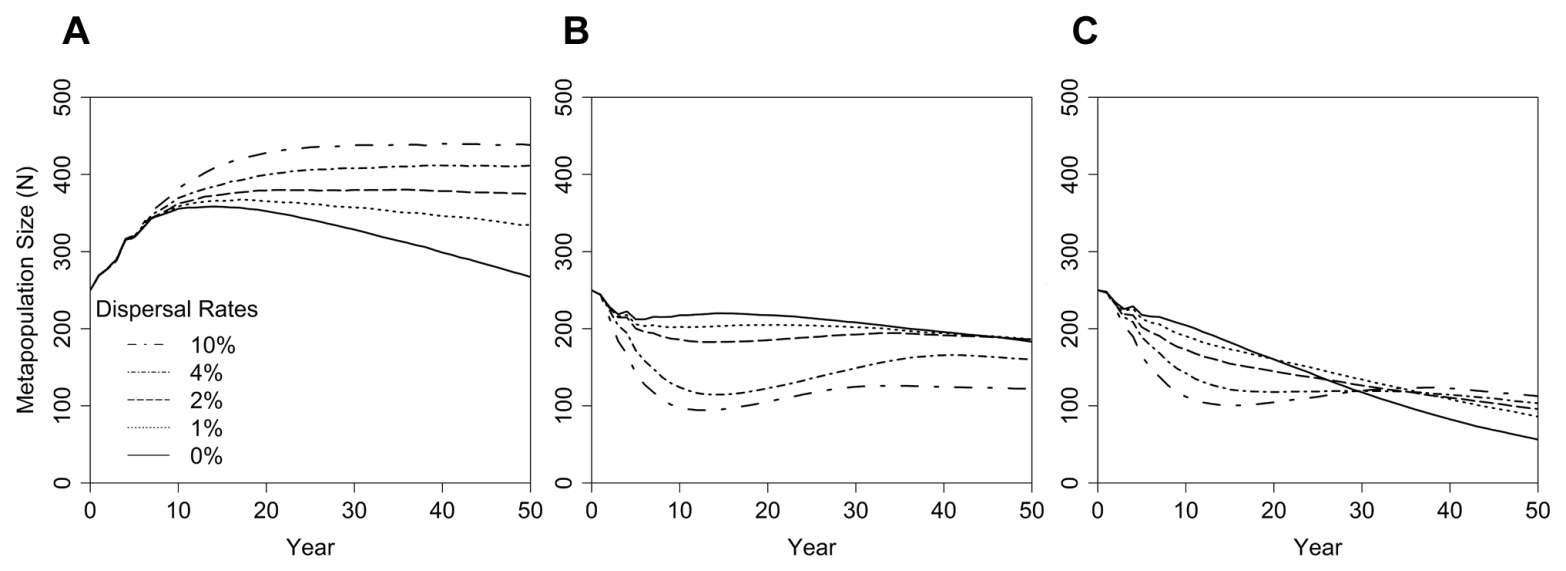
Metapopulation dynamics influenced by dispersal. Metapopulation size is projected by (A) a PVA model in *Vortex* assuming varied rates of dispersal, annual fluctuations in demographic rates, and inbreeding depression; (B) a metamodel that linked a PVA model in *Vortex* to an infectious disease model in *Outbreak*, assuming varied rates of dispersal, minimal annual fluctuations in demographic rates, and no inbreeding depression; and (C) a metamodel that linked a PVA in *Vortex* to an infectious disease model in *Outbreak*, assuming varied rates of dispersal, annual fluctuations in demographic rates, and inbreeding depression. In (A), higher rates of dispersal increase growth and stability of the metapopulation because stochastic effects in local subpopulations are dampened. When disease was introduced but stochasticity was removed, as in (B), higher rates of dispersal depress population size because of the faster spread of disease. Finally, when stochasticity, disease, and dispersal were considered in (C), higher dispersal initially reduced population size because of the faster spread of disease. In later years, disease was largely eliminated from the system, and higher rates of dispersal stabilized the population against stochastic fluctuations. During a few years in the middle of the simulation, disease and stochastic processes were equally important, and intermediate rates of dispersal led to the highest population size.

In the metapopulation PVA model alone, with no disease, increasing dispersal led to larger and more sustained population growth (Figure 3a). With no or little dispersal among sub-populations, each small sub-population was vulnerable to stochastic fluctuations that resulted in depressed mean population growth, inbreeding depression, and, ultimately, population decline after about 15 years. When dispersal rates exceeded 1% per year, destabilizing effects of population fragmentation were mostly countered as demonstrated by the absence of long-term population decline. 

#### Impacts of connectivity on spread of disease

In the mostly disease-driven model, dispersal had the opposite effect on metapopulation size (Figure 3b). Infectious disease caused immediate declines in metapopulation size. This decline was severe under high dispersal as disease more rapidly spread among all sub-populations. After about 10 years, the infectious disease had largely disappeared from the metapopulation as individuals recovered and became resistant and as smaller population sizes were less able to sustain the epidemic. However, occasional re-infection of the metapopulation from outside sources, coupled with renewed rapid spread of the disease, caused the mean metapopulation size to remain low in scenarios with high rates of dispersal. Sub-populations with the lowest levels of dispersal avoided the sharp disease-driven declines because of the slow spread of disease. The negative effects of fragmentation seen in the disease-free metapopulation (Figure 3a) were reduced and delayed because lower stochasticity in the disease-driven model helped to stabilize the isolated subpopulations and because inbreeding impacts were not included. 

Differences in the impacts of population connectivity on metapopulation dynamics in the absence (Figure 3a) or presence (Figure 3b) of disease illustrates the opposing forces acting on sub-divided populations: stochastic processes threaten small, isolated populations and are countered by dispersal while infectious disease transmission threatens inter-connected populations and is blocked by isolation. Both processes impact many real populations, but the balance between these forces would be difficult to predict from the application of standard PVA methodology for population modeling, from standard epidemiological models of disease, or even from the examination of both models independently, as illustrated above. 

#### Metamodel analysis of the dual effects of stochasticity and disease

The results of the full metamodel with both stochastic demography and disease demonstrated population trends that reflected the complex interaction between disease spread at high dispersal rates and population decline due to demographic stochasticity and inbreeding at low dispersal rates (Figure 3c). The synergistic effects of stochastic processes and epidemic disease resulted in population dynamics driven initially by disease but later by small population fluctuations. Until year 20, increasing dispersal led to lower metapopulation size because of the more rapid spread of disease; the pattern was reversed beyond year 35, and increasing dispersal led to greater metapopulation size because of the stabilization of demographic fluctuations. Between simulation years 20 through 35, intermediate dispersal rates resulted in the highest metapopulation sizes; the partial benefits of connectivity reduced inbreeding and other damaging effects of fragmentation, but dispersal was low enough to slow the spread of infectious disease. 

Overall, the balance between positive and negative effects of population fragmentation was dependent on the temporal scale over which metapopulation dynamics were projected as well as the specific population and disease parameters applied in the metamodel. Changes to population size, intrinsic rates of population growth, annual variation in demographic rates, severity of inbreeding depression, or transmissibility and pathogenicity of disease could have changed metamodel predictions regarding the range of dispersal rates that would lead to greatest metapopulation viability. Metamodels will perhaps be most useful for investigations of the compound threats facing specific populations in specific circumstances precisely because outcomes are determined by complex interactions that are sometimes synergistic and sometimes countervailing. 

### Case Study II

#### Single-species PVA projections

**Figure 4:**
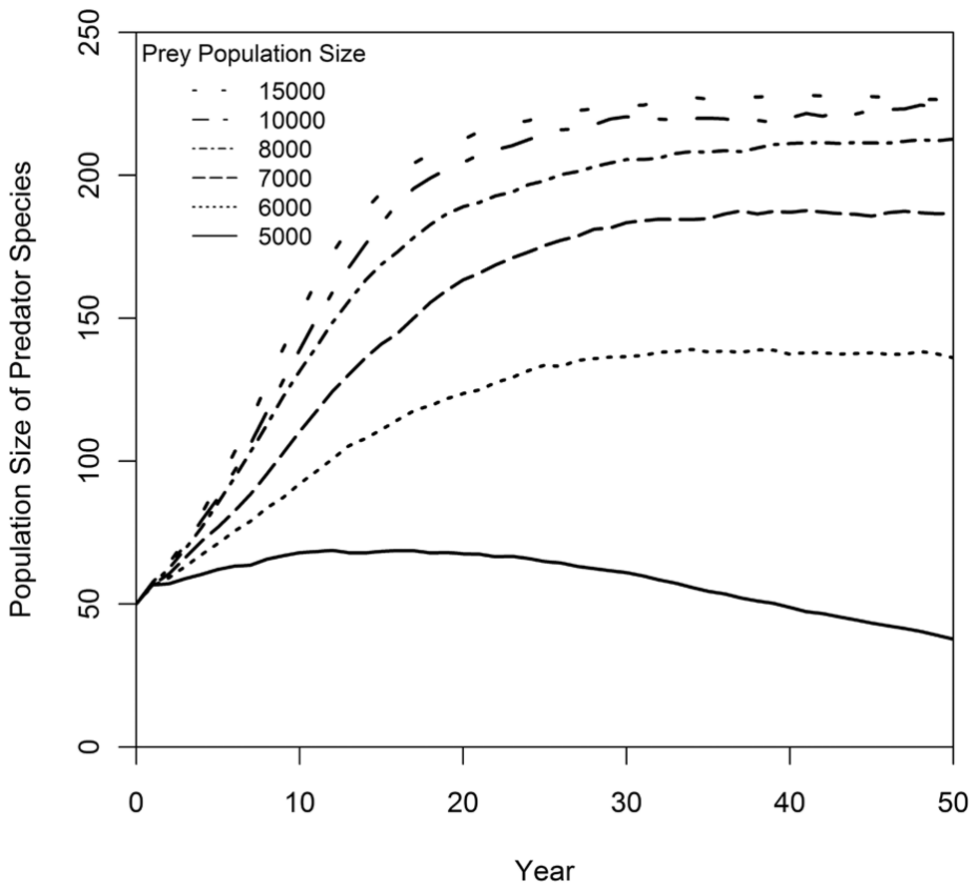
Population trajectories for a predator species at different levels of prey availability. Mean predator population size through time is predicted by a single-species PVA model that assumed a fixed prey population size. Simulations were run for prey populations of 5000, 6000, 7000, 8000, 10000, and 15000 individuals. Approximately 6000 prey was sufficient to sustain growth of the predator population from its initial N = 50 to more than 100.

**Figure 5:**
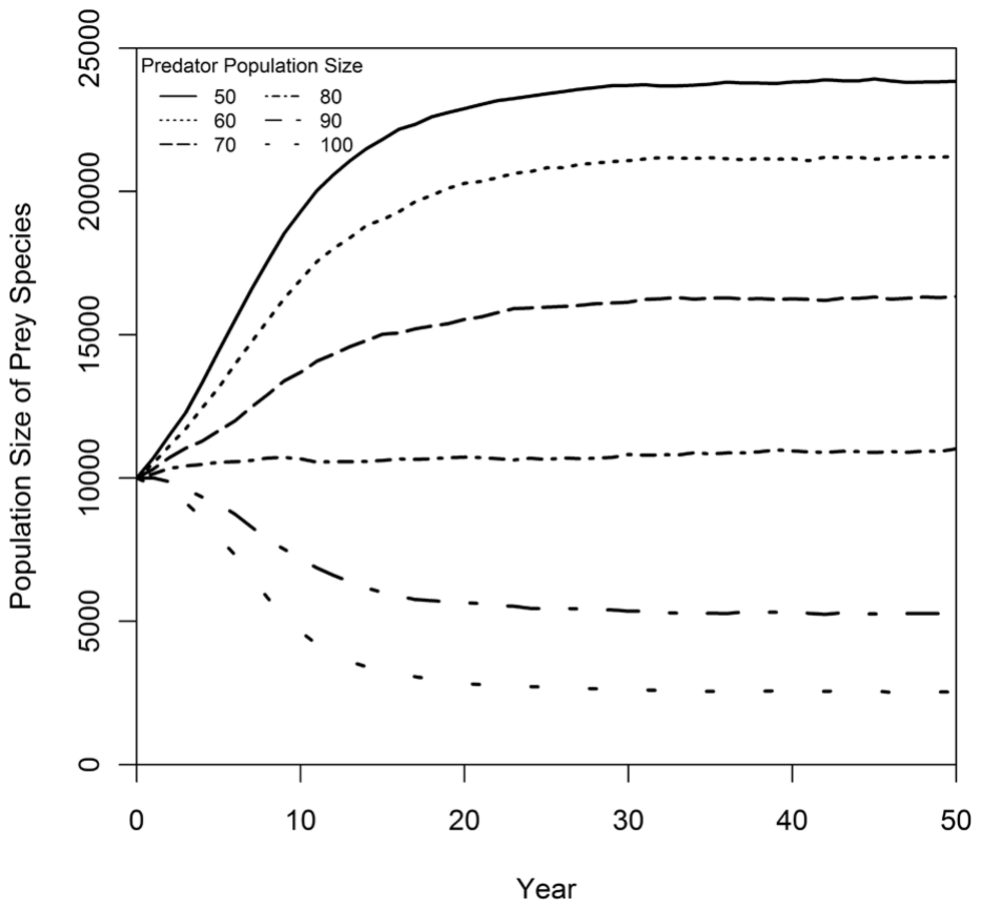
Population trajectories for a prey species subjected to different levels of predation. Mean prey population size through time is predicted by a single-species PVA model that assumed a fixed predator population size. Simulations were run for predator populations of 50, 60, 70, 80, and 100 individuals. The prey population was sustained at a size of N = 10000 or more if there were 80 or fewer predators.

The population dynamics of a predator and a prey species were examined first in separate PVA models with the assumption that the other species was a constant factor (determinant of reproduction for the predator; determinant of mortality for the prey). With a fixed size prey population of N(prey) = 10000, the predator population was sustained with an average growth rate of r = 0.108 as it approached its carrying capacity. Models with prey populations from N(prey) = 5000 to N(prey) = 15000 indicated that, when N(prey) > 6000, the predator population was sustained and remained near K(predator) (Figure 4). Similarly, with a constant number of predators, N(predator) = 50, the prey population could sustain the added mortality and grew initially at a rate of r = 0.055 with an accelerated rate of r = 0.109 (due to a lower ratio of predators to prey) as the population approached K(prey). The prey species achieved positive population growth whenever N(predator) < 80 (Figure 5). Thus, the single species PVA models predicted stable single-species dynamics in the predator-prey system; sufficient prey were available to support the predator population, and the predation rate was sustainable for the prey. 

#### Predator-prey dynamics in the absence of external sources of stochasticity

**Figure 6:**
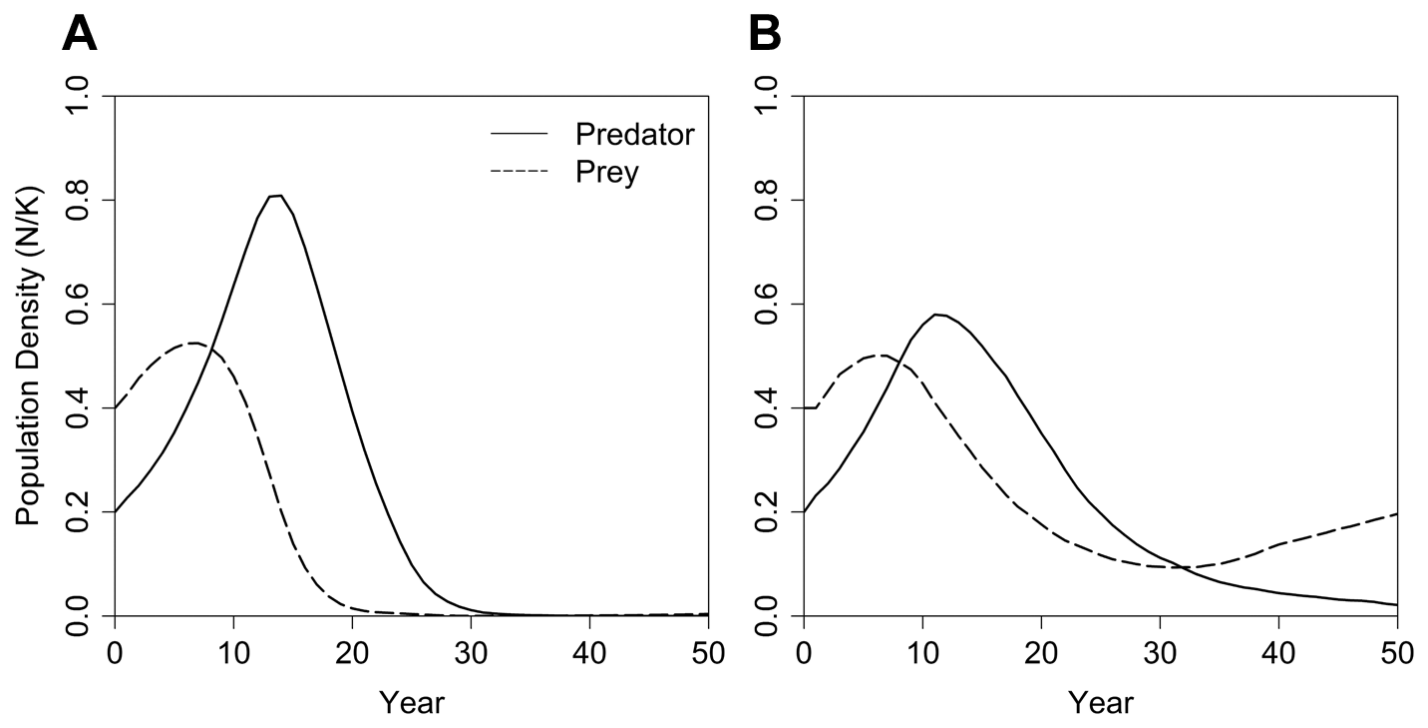
Mean predator-prey dynamics in coupled metamodels that (A) did not include and (B) did include stochastic variation. Mean population densities (N/K) for a predator and a prey species are predicted by a two-species metamodel, which assumed that the density of one species would impact the other species. In (A), externally driven sources of stochasticity (e.g., environmental variation, catastrophes) and inbreeding did not impact either population, and we found that the predator population grew rapidly, causing collapse of the prey population followed by collapse of the predator population. In (B), externally driven stochasticity and inbreeding depression could impact each population. For this scenario, the average trajectory shows that the predator population grew, followed by a decline in prey, causing subsequent decline in the predator, eventually resulting in a possibly stable state in which a reduced prey population sustained a reduced predator population.

The PVA models were then coupled to investigate dynamics when changes in the number of each species simultaneously affect the other. The dynamics that could arise from analytical models, such as coupled deterministic population growth models, were approximated by removing stochasticity other than the demographic variation intrinsic to individual-based PVA models. For this test, there was no environmental variation in demographic rates across years, mean effects of catastrophes were averaged across years, and inbreeding depression was removed. This resulted in a tight coupling of predator-prey dynamics with an initial rise in the prey population followed closely by a rise in the predator population. This caused a rapid collapse of the over-consumed prey followed by a collapse of the predator population in the absence of the prey (Figure 6a). 

#### Full metamodel dynamics of predator-prey interactions with external sources of stochasticity

**Figure 7:**
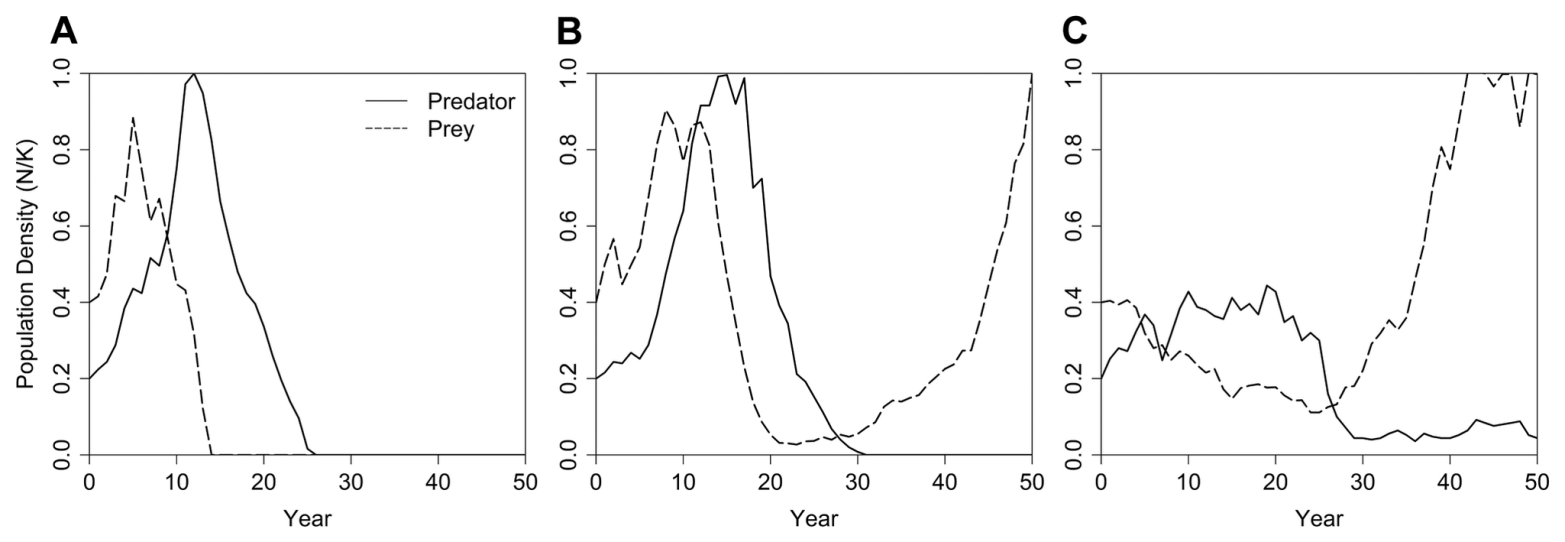
Individual trajectories of predator and prey populations in a coupled metamodel subjected to stochastic variation. Simulated dynamics of the two-species predator-prey metamodel showed several different patterns for independent iterations. Examples are shown for iterations that exhibited patterns where (A) both predator and prey populations initially increased but then collapsed, (B) the predator population went extinct while there were still enough prey individuals to allow for recovery, and (C) both predator and prey persisted with dynamics that suggested that there could be various, possibly unstable, equilibria.

The linked predator-prey system, when each species was also subjected to the kinds of stochasticity that are often modeled in PVAs (e.g., environmental variation in demographic rates, catastrophes, inbreeding depression), generated mean trajectories that suggested that a stable predator-prey system with a reduced prey population sustaining a small population of the predator could arise after an initial increase and then decrease of both populations (Figure 6b). However, the simulated dynamics followed any of several different patterns in the independent iterations. Consequently, the mean population trajectories averaged across iterations (Figure 6b) obscured dynamics that could be observed in individual simulations (Figure 7). In many iterations, both populations followed the pattern seen in the absence of externally driven stochasticity (Figure 6a): a rapid rise in population size for the prey and predator, followed by a collapse and, ultimately, extinction of both populations (Figure 7a). In other iterations, the predator population went extinct while enough prey individuals remained to allow for recovery (Figure 7b). In yet other cases, both predator and prey persisted with dynamics that suggested that there could be various, possibly unstable, equilibria – such as a reduced prey base sustaining a predator population below its carrying capacity or an inbred predator population persisting on an abundant prey (Figure 7c). 

This implementation of a two-species metamodel revealed multiple possible outcomes that were not generated by the separate single-species PVAs (each predicting growth and stability of their focal species) or from the more deterministic predator-prey coupling (predicting collapse of both species). Thus, PVA projections that presume that strong interactions with other species can be adequately represented by mean trends should be viewed with caution. Similarly, analytical or simple simulation models that presume that feedbacks between species models can ignore random stochastic forces that might push species interactions into different zones of dynamic behavior should be met with skepticism. Other sets of parameters defining life histories, relationships between species, or external factors could well have led to different or more predictable results, but the first array of parameters that we chose to test led to these surprising findings. The power of the metamodel approach is that it allows full exploration of the effects of model structure, functional relationships, and parameter values. A more extensive multi-species metamodel could explore effects of additional species in the system, such as alternative prey, competing predators, or multiple trophic levels. 

## Discussion

The concept of a metamodel has been exploited in other fields to create more powerful and flexible representations of systems. A metamodel framework (termed “federated architecture”) has been developed to integrate neuronal simulation software [57]. LANDIS [58,59] is a metamodel for simulating changes in forest structure and composition, providing a platform for linking components modeling forest succession, disturbance, climate change, and seed dispersal, with options for further extensions by a user community. BioMove [60] is a metamodel for simulating range-shifts in plant populations, in which users can combine models of landscape configuration, habitat characteristics, population demography, and dispersal. ATLSS [61] is a series of spatially explicit species models (termed a “multimodel” by its developers) for the Everglades ecosystem that use common modeling tools and can be driven by linked representations of habitat characteristics (e.g., hydrology and vegetation patterns). We conceptualize metamodels as even more flexible and broadly applicable than the tools available to date for ecological modeling, and we developed *MetaModel Manager* as a generic platform for extending PVA through the use of metamodel links. Importantly, the linked models can be programmed in any language that can compile Windows Dynamic-Link Libraries. Thus, with the addition of small accessor functions, almost any existing or new Windows-based model of ecological processes can be integrated with a PVA. 

The two hypothetical examples presented in the Results show that metamodels can reveal patterns not predicted by the component models alone. Investigations of the dynamics of real wildlife populations should include extensive sensitivity testing to evaluate the impacts of model structure and parameter uncertainty. Even more so than with standard PVA models, the number of factors in a metamodel that are uncertain or potentially subject to manipulation will typically be large, making an exploration of all factorial combinations impractical. One approach for sensitivity testing of many potentially interacting variables is to generate a large number of scenarios, each with parameters sampled from the distributions describing the ranges of uncertainty. Regression analyses can then be used to test the impact of each factor (including interactions) on model predictions [62–64]. 

### Cases Being Explored with Metamodels

Several metamodels implemented with *MetaModel Manager* have recently been published. Keet et al. [65] linked a model of socially structured population dynamics of lions in Kruger National Park (*SimSimba* [66,67]) to an *Outbreak* model of bovine tuberculosis transmission among lions and their prey. The metamodel was complex and novel in that it included feedbacks between the lion social system and disease epidemiology such that the social system impacted the spread of the disease and the disease disrupted the social system. Similarly, Bradshaw et al. [68] linked an *Outbreak* model of bovine tuberculosis in feral buffalo in Australia to a *Vortex* model of population dynamics to explore efficacy of disease surveillance and management strategies. Prowse et al. [69] used a metamodel to explore functional responses among trophic levels in an investigation of the possible causes of the thylacine extinction from Tasmania following European settlement. Their metamodels included an analytical model of vegetation growth in response to rainfall and herbivore density; numerical responses of macropods to vegetative biomass; time series data on sheep as competitors of macropods for vegetation; impacts of prey availability on thylacine demography; decline in Aboriginal hunting of macropods as a competitor of thylacines; harvesting of both macropods and thylacine by Europeans; and habitat conversion by Europeans. From the metamodels, they concluded that the rapid extinction of the thylacine could have been due to the synergistic effects of these forces without needing to invoke disease or other unobserved factors.

With various sets of collaborators, investigations are underway to test metamodel linkages among other models relating to PVA (*RAMAS* [55]), animal dispersal (*Spatial* [54]), landscape change (*TELSA* [70]), and climate change projections (e.g., extending the work in Brook et al. [71]). A topic of ongoing debate that might benefit from exploration with a metamodel approach is the study of population cycles. Cycles have been attributed to predator-prey systems with delays, to fluctuations in food supply, to dispersal behaviors, to weather patterns, to diseases, and to other causes (e.g., [72–75]) as well as being ascribed to spurious outcomes of stochastic fluctuations. Metamodels provide a tool to explore the outcomes of interactions among predator-prey-vegetation trophic dynamics, dispersal patterns, disease, and external drivers such as weather – all within stochastically variable systems. 

### Advantages and Disadvantages of Increasing Model Complexity

With the use of models in any discipline, trade-offs are inevitable between model simplicity (providing clearer insight into important factors) and model complexity (providing a more complete representation of the interacting array of factors). The metamodel approach heightens the need to manage these trade-offs carefully. Perhaps the biggest challenge to using metamodels is describing the functional linkages between models. In some cases, the impacts of the dynamics of one model on another are implicit and emergent. For example, the coupling of an epidemiological model to a stochastic demographic model can reveal the effects that demographic fluctuations would have on the spread of disease, even if no parameter other than population size was passed between models and neither model incorporated feedback from the other model into its own parameterization. However, for many other metamodels (e.g., predator-prey models, social system dynamics, and projections incorporating climate change), the functional relationships through which outputs from one model serve as necessary inputs to the next must be specified via functions that use transferred variables to drive other processes. 

We hypothesize that the use of metamodels will reveal emergent properties in ecological systems that would not have been observed if component processes were analyzed in isolation. For example, many epidemiological models of infectious disease assume that the host population is constant in size, experiencing simple exponential growth, or driven by disease dynamics. Yet, the maintenance and spread of an epidemic can have different dynamics if the host population is subject to large fluctuations in size and distribution unrelated to the disease itself [76]. Conversely, most PVA models treat disease as a constant or simple stochastic source of mortality (if disease is considered at all), but the impact of disease on population viability can be quite different when disease occurs in episodic outbreaks that spread through a population. Consequently, predictions from metamodels should be more accurate and robust, as perturbations to otherwise static factors can be explicitly modeled. 

However, it is also possible that the use of metamodels will not improve understanding and management of a complex ecological system. First, as with any model, if the factors entered into the metamodel are not those that principally drive true system dynamics, then the model will not represent the natural world. The metamodel approach was pursued specifically to reduce the frequency with which important processes are ignored, but it is still vulnerable to the problem that practitioners tend to rely on the models and data with which they are familiar. If the ability to link a few models gives practitioners inappropriately false confidence that all the important parts of a complex system are understood, then the metamodel will have perversely counteracted its purpose to encourage broader consideration of pressures impinging on the system. For this reason, it will be even more important that metamodel analyses include sensitivity testing, including examinations of: (1) including or omitting component models, (2) changing descriptions of the functional linkages between subsystems, (3) including or omitting specific threats, and (4) altering parameter values. Structural uncertainty in the processes driving the system becomes a more significant concern when more processes are included in the overall analysis. Nevertheless, metamodels provide a means to reduce the false confidence that can result when many processes are ignored to keep an analysis comfortably within one discipline or realm of expertise.

### Implications for the Development of Integrated, Science-Based Conservation Strategies

When used well, metamodels can balance the partitioning of highly complex systems into simpler component parts while preserving the ability to represent the emergent effects of interactions among those components. This novel framework provides a means of moving beyond population risk assessments that only consider impacts of isolated threats. The metamodel approach will allow for more realistic models that can guide management, test interactions among diverse threats, and include additional expertise in comprehensive population analyses.

The characteristics that define a well-functioning metamodel are parallel to principles that promote collaboration among people working on a shared problem. These include: solicitation of experts from diverse disciplines; open sharing of data; documentation of individual and synthetic outputs; moderated sequential participation in modifying and expanding information; translation as needed among the technical languages; and openness to the addition of new participants and types of data [77–79]. Indeed, the ideas for the programming concepts and implementation of *MetaModel Manager* software arose from discussions among natural and social scientists about methods for transdisciplinary collaboration [20,22,33]. Practitioners of disciplines necessary for risk assessments of populations (e.g., ecologists, geneticists, wildlife veterinarians, land managers, and resource users) often work in isolation from experts in the other fields, rarely sharing information effectively and even more rarely collaborating on analyses and planning. A metamodel provides a methodology that is as much social as it is technical by allowing people across multiple disciplines to integrate their knowledge into a comprehensive representation of the issues and options.

## References

[1] Liu J, Dietz T, Carpenter SR, Alberti M, Folke C et al. (2007) Complexity of coupled human and natural systems. Science 317: 1513-1516. doi:10.1126/science.1144004. PubMed: 17872436. 17872436

[2] Beissinger SR, Westphal MI (1998) On the use of demographic models of population viability in endangered species management. J Wildl Manage 62: 821-841. doi:10.2307/3802534.

[3] Fieberg J, Ellner SP (2001) Stochastic matrix models for conservation and management: a comparative review of methods. Ecol Lett 4: 244–266. doi:10.1046/j.1461-0248.2001.00202.x.

[4] Morgan MS, Morrison M, eds. (1999) Models as Mediators: Perspectives on Natural and Social Science. Cambridge: Cambridge University Press. 420 p.

[5] Morris WF, Doak DF (2002) Quantitative Conservation Biology. Theory and Practice of Population Viability Analysis. Sunderland, MA: Sinauer. 480 p.

[6] Lacy RC (2000) Considering threats to the viability of small populations. Ecol Bull 48: 39-51.

[7] Melbourne BA, Hastings A (2008) Extinction risk depends strongly on factors contributing to stochasticity. Nature 454: 100-103. doi:10.1038/nature06922. PubMed: 18596809. 18596809

[8] Lindenmayer DB, Lacy RC, Pope ML (2000) Testing a simulation model for population viability analysis. Ecol Appl 10: 580-597. Available online at: doi:10.1890/1051-0761(2000)010[0580:TASMFP]2.0.CO;2

[9] Lindenmayer DB, Possingham HP, Lacy RC, McCarthy MA, Pope ML (2003) How accurate are population models? Lessons from landscape-scale tests in a fragmented system. Ecol Lett 6: 41-47.

[10] Stephens P, Frey-Roos F, Arnold W, Sutherland W (2002) Model complexity and population predictions: the alpine marmot as a case study. J Anim Ecol 71: 343-361. doi:10.1046/j.1365-2656.2002.00605.x.

[11] Vucetich JA, Peterson RO, Waite TA (1997) Effects of social structure and prey dynamics on extinction risk in gray wolves. Conserv Biol 11: 957–965. doi:10.1046/j.1523-1739.1997.95366.x.

[12] Vucetich JA, Creel S (1999) Ecological interactions, social organization, and extinction risk in African wild dogs. Conserv Biol 13: 1172–1182. doi:10.1046/j.1523-1739.1999.98366.x.

[13] Gerber L (2006) Including behavioral data in demographic models improves estimates of population viability. Front Ecol Environ 4: 419-427. Available online at: doi:10.1890/1540-9295(2006)4[419:IBDIDM]2.0.CO;2

[14] Allendorf FW, Ryman N (2002) The role of genetics in population viability analysis. In: Beissinger SR McCullough DR Population Viability Analysis. Chicago: University of Chicago Press pp. 50-85.

[15] Brook BW, Sodhi NS, Bradshaw CJA (2008) Synergies among extinction drivers under global change. Trends Ecol Evol 23: 453-460. doi:10.1016/j.tree.2008.03.011. PubMed: 18582986. 18582986

[16] Lawler J, Campbell S, Guerry A, Kolozsvary M, O'Connor R et al. (2002) The scope and treatment of threats in endangered species recovery plans. Ecol Appl 12: 663-667. Available online at: doi:10.1890/1051-0761(2002)012[0663:TSATOT]2.0.CO;2

[17] Munns WR Jr (2006) Assessing risks to wildlife populations from multiple stressors: overview of the problem and research needs. Ecol Soc 11: 23 Available: http://www.ecologyandsociety.org/vol11/iss1/art23/. Accessed 2013 November 24

[18] Schemske D, Husband B, Ruckelshaus M, Goodwillie C, Parker J et al. (1994) Evaluating approaches to the conservation of rare and endangered plants. Ecology 75: 584-606. doi:10.2307/1941718.

[19] Carroll R, Augspurger C, Dobson A, Franklin J, Orians G et al. (1996) Strengthening the use of science in achieving the goals of the Endangered Species Act: an assessment by the Ecological Society of America. Ecol Appl 6: 1-11. doi:10.2307/2269537.

[20] Nyhus PJ, Westley FR, Lacy RC, Miller PS (2002) A role for natural resource social science in biodiversity risk assessment. Soc Nat Resour 15: 923-932. doi:10.1080/08941920290107657.

[21] Miller PS, Lacy RC (2003) Integrating the human dimension into endangered species risk assessment. In: Westley FR Miller PS Experiments in Consilience: Integrating Social and Scientific Responses to Save Endangered Species. Washington, DC: Island Press pp. 41-63.

[22] Westley FR, Miller PS eds. (2003) Experiments in Consilience: Integrating Social and Scientific Responses to Save Endangered Species. Washington, DC: Island Press. 393 p.

[23] Sabo JL (2008) Population viability and species interactions: Life outside the single-species vacuum. Biol Conserv 141: 276-286. doi:10.1016/j.biocon.2007.10.002.

[24] Traill LW, Lim MLM, Sodhi NS, Bradshaw CJA (2010) Mechanisms driving change: altered species interactions and ecosystem functions from global warming. J Anim Ecol 79: 937-947. doi:10.1111/j.1365-2656.2010.01695.x. PubMed: 20487086. 20487086

[25] Chapin FS III, Robards MD, Huntington HP, Johnstone JF, Trainor SF et al. (2006) Directional changes in ecological communities and social-ecological systems: A framework for prediction based on Alaskan examples. Am Nat 168: S36-S49. doi:10.1086/509047. PubMed: 17109327. 17109327

[26] Cressie N, Calder CA, Clark JS, Ver Hoef JM, Wikle CK (2009) Accounting for uncertainty in ecological analysis: the strengths and limitations of hierarchical statistical modeling. Ecol Appl 19: 553-570. doi:10.1890/07-0744.1. PubMed: 19425416. 19425416

[27] Burgman M, Franklin J, Hayes KR, Hosack GR, Peters GW et al. (2012) Modeling extreme risks in ecology. Risk Anal 32: 1956-1966. doi:10.1111/j.1539-6924.2012.01871.x. PubMed: 22817845. 22817845

[28] Lacy RC (1993/1994) What is Population (and Habitat) Viability Analysis? Primate Conservation 14/15: 27-33.

[29] Westley FR, Byers O (2003) Getting the right science and getting the science right: Process design and facilitation in PHVA workshops. In: Westley FR Miller PS Experiments in Consilience: Integrating Social and Scientific Responses to Save Endangered Species. Washington, DC: Island Press pp. 64-82.

[30] IUCN / SSC (2008) Strategic Planning for Species Conservation: A Handbook, Version 1.0. Gland, Switzerland: IUCN Species Survival Commission. 104 p.

[31] Redford KH, Amato G, Baillie J, Beldomenico P, Bennett EL et al. (2011) What does it mean to successfully conserve a (vertebrate) species. BioScience 61: 39-48.

[32] Miller PS, Lacy RC (2003) Metamodels as a tool for risk assessment. In: Westley FR Miller PS Experiments in Consilience: Integrating Social and Scientific Responses to Save Endangered Species. Washington, DC: Island Press pp. 333-351.

[33] Nyhus PJ, Lacy R, Westley FR, Miller PS, Vredenburg H, et al. (2007) Tackling biocomplexity and meta-models for species risk assessment. Ecol Soc 12: 31 Available: http://www.ecologyandsociety.org/vol12/iss1/art31/. Accessed 2013 November 24

[34] Holling CS (1996) Surprise for science, resilience for ecosystems, and incentives for people. Ecol Appl 6: 733-735. doi:10.2307/2269475.

[35] Holling CS (1998) Two cultures of ecology. Conservation Ecology 2: 4 Available: http://www.consecol.org/vol2/iss2/art4/. Accessed 2013 November 24

[36] Nicholson CR, Starfield AM, Kofinas GP, Kruse JA (2002) Ten heuristics for interdisciplinary modeling projects. Ecosystems 5: 376-384. doi:10.1007/s10021-001-0081-5.

[37] Lacy RC, Lindenmayer DB (1995) A simulation study of the impacts of population subdivision on the mountain brushtail possum, *Trichosurus* *caninus* Ogilby (Phalangeridae: Marsupialia), in south-eastern Australia. II. Loss of genetic variation within and between subpopulations. Biol Conserv 73: 131-142. doi:10.1016/0006-3207(95)91940-Y.

[38] Lindenmayer DB, Lacy RC (1995) A simulation study of the impacts of population subdivision on the mountain brushtail possum, *Trichosurus* *caninus* Ogilby (Phalangeridae: Marsupialia), in south-eastern Australia. I. Demographic stability and population persistence. Biol Conserv 73: 119-129. doi:10.1016/0006-3207(95)00049-A.

[39] Fahrig L (2002) Effects of habitat fragmentation on the extinction threshold: A synthesis. Ecol Appl 12: 346-353. doi:10.2307/3060946.

[40] Dobson AP, May RM (1986) Disease and conservation. In: Soulé ME Conservation Biology. The Science of Scarcity and Diversity. Sunderland, MA: Sinauer pp. 345-365.

[41] Hess G (1996) Disease in metapopulation models: Implications for conservation. Ecology 77: 1617-1632. doi:10.2307/2265556.

[42] Vanclay JK, Sinclair FL, Prabhu R (2003) Modelling interactions amongst people and forest resources at the landscape scale. Small-Scale Forestry 2: 117-120.

[43] Schumaker NH http://www.epa.gov/wed/pages/models/hexsim/index.htm.

[44] Willis KJ, Bhagwat SA (2009) Biodiversity and climate change. Science 326: 806-807. doi:10.1126/science.1178838. PubMed: 19892969. 19892969

[45] Purves D, Scharlemann J, Harfoot M, Newbold T, Tittensor DP et al. (2013) Time to model all life on earth. Nature 493: 295-297. PubMed: 23325192. 23325192 10.1038/493295a

[46] Keith DA, Akçakaya HR, Thuiller W, Midgley GF, Pearson RG et al. (2008) Predicting extinction risks under climate change: coupling stochastic population models with dynamic bioclimatic habitat models. Biol Lett 4: 560–563. doi:10.1098/rsbl.2008.0049. PubMed: 18664424. 18664424 PMC2610061

[47] Anderson BJ, Akçakaya HR, Araújo MB, Fordham DA, Martinez-Meyer E et al. (2009) Dynamics of range margins for metapoulations under climate change. Proc R Soc of London B 276: 1415-1420. doi:10.1098/rspb.2008.1681. PubMed: 19324811. PMC2677226 19324811

[48] Pollak JP, Lacy RC (2013) MetaModel Manager, Version 1.0. Brookfield, Illinois: Chicago Zoological Society Available: http://www.vortex10.org/MeMoMa.aspx. Accessed 2013 November 24

[49] Raboy BE, Lacy RC (2013) MetaModel Manager user’s manual. Brookfield, IL: Chicago Zoological Society Available: http://www.vortex10.org/MeMoMa.aspx. Accessed 2013 November 24

[50] Hunter CM, Caswell H, Runge MC, Regehr EV, Amstrup SC et al. (2010) Climate change threatens polar bear populations: a stochastic demographic analysis. Ecology 91: 2883-2897. doi:10.1890/09-1641.1. PubMed: 21058549. 21058549

[51] Lacy RC (2000) Structure of the VORTEX simulation model for population viability analysis. Ecol Bull 48: 191-203.

[52] Lacy RC, Pollak JP (2013) VORTEX: A Stochastic Simulation of the Extinction Process, Version 10.0. Brookfield, Illinois: Chicago Zoological Society Available: http://www.vortex10.org/Vortex10.aspx. Accessed 2013 November 24

[53] Lacy RC, Pollak JP, Miller PS, Hungerford L, Bright P (2012) *Outbreak* version 2.0. Apple Valley, MN: IUCN SSC Conservation Breeding Specialist Group Available: http://www.vortex10.org/Outbreak.aspx. Accessed 2013 November 24

[54] Pollak JP (2013) Spatial model of animal movement on landscapes, version 1.0. New York: JP Pollak Available: http://www.vortex10.org/Spatial.aspx. Accessed 2013 November 24

[55] Akçakaya HR (2009) RAMAS Metapop: viability analysis for stage-structured metapopulations (version 6.0). Setauket, NY: Applied Biomathematics.

[56] Morton NE, Crow JF, Muller HJ (1956) An estimate of the mutational damage in man from data on consanguineous marriages. Proc Natl Acad Sci U S A 42: 855-863. doi:10.1073/pnas.42.11.855. PubMed: 16589958. 16589958 PMC528351

[57] Cornelis H, Coop AD, Bower JM (2013) A Federated Design for a Neurobiological Simulation Engine: The CBI Federated Software Architecture. PLOS ONE 7(1): e28956. doi:10.1371/journal.pone.0022856. PMC3252298 22242154

[58] Scheller RM, Mladenoff DJ (2007) Forest landscape simulation models: Tools and strategies for projecting and undertanding spatially extensive forest ecosystems. Landscape Ecol 22: 491-505. doi:10.1007/s10980-006-9048-4.

[59] Scheller RM, Domingo JB, Sturtevant BR, Williams JS, Rudy A et al. (2007) Design, development, and application of LANDIS-II, a spatial landscape simulation model with flexible spatial and temporal resolution. Ecol Modelling 201: 409-419. doi:10.1016/j.ecolmodel.2006.10.009.

[60] Midgley GF, Davies ID, Albert CH, Altwegg R, Hannah L et al. (2010) BioMove – an integrated platform simulating the dynamic response of species to environmental change. Ecography 33: 612-616.

[61] Gross LJ, DeAngelis DL (2002) Multimodeling: new approaches for linking ecological models. In: Scott JM Heglund PJ Morrison M Raphael M Haufler J Predicting species occurrences: Issues of scale and accuracy. Covello, CA: Island Press . pp. 467-474

[62] McCarthy M, Burgman M, Ferson S (1995) Sensitivity analysis for models of population viability. Biol Conserv 73: 93-100. doi:10.1016/0006-3207(95)00046-7.

[63] McCarthy M, Burgman M, Ferson S (1996) Logistic sensitivity and bounds for extinction risks. Ecol Modelling 86: 297-303. doi:10.1016/0304-3800(95)00067-4.

[64] Cross PC, Beissinger S (2001) Using logistic regression to analyze the sensitivity of PVA models: A comparison of methods based on African wild dog models. Conserv Biol 15: 1335-1346. doi:10.1046/j.1523-1739.2001.00031.x.

[65] Keet DF, Davies-Mostert H, Bengis RG, Funston P, Buss P, et al. (2009). Disease Risk Assessment Workshop Report: African Lion (Panthera leo) Bovine Tuberculosis. Conservation Breeding Specialist Group (CBSG SSC / IUCN) / CBSG Southern Africa. Endangered Wildlife Trust.

[66] Quadling HS, Starfield AM (2002) Exploiting object-orientated programming structures in the quest for an individual-based lion population model with an attractive user interface. S Afr J Sci 98: 449-454.

[67] Whitman KL, Starfield AM, Quadling H, Packer C (2007) Modelling the effects of trophy selection and environmental disturbance on a simulated population of African lions. Conserv Biol 21: 591-601. doi:10.1111/j.1523-1739.2007.00700.x. PubMed: 17531038. 17531038

[68] Bradshaw CJA, McMahon CR, Miller PS, Lacy RC, Watts MJ et al. (2012) Novel coupling of individual-based epidemiological and demographic models predicts realistic dynamics of tuberculosis in alien buffalo. J Appl Ecol 49: 268-277. doi:10.1111/j.1365-2664.2011.02081.x.

[69] Prowse TAA, Johnson CN, Lacy RC, Bradshaw CJA, Pollak JP et al. (2013) No need for disease: testing extinction hypotheses for the thylacine using multi-species metamodels. J Anim Ecol 82: 355-364. doi:10.1111/1365-2656.12029. PubMed: 23347431. 23347431

[70] Kurz W, Beukema S, Klenner W, Greenough J, Robinson D et al. (2000) TELSA: The tool for exploratory landscape scenario analyses. Comput Electron Agric 27: 227-242. doi:10.1016/S0168-1699(00)00109-5.

[71] Brook BW, Akçakaya HR, Keith DA, Mace GM, Pearson RG et al. (2009) Integrating bioclimate with population models to improve forecasts of species extinctions under climate change. Biol Lett 5: 723-725. doi:10.1098/rsbl.2009.0480. PubMed: 19625300. 19625300 PMC2828003

[72] Krebs CJ (2008) Ecology: The Experimental Analysis of Distribution and Abundance, 6th ed. Menlo Park, CA: Benjamin/Cummings. 688 p.

[73] Krebs CJ, Boonstra R, Boutin S, Sinclair ARE (2001) What drives the 10-year cycle of snowshoe hares? Bioscience 51: 25-35. Available online at: doi:10.1641/0006-3568(2001)051[0025:WDTYCO]2.0.CO;2

[74] Korpimaki E, Brown PR, Jacob J, Pech RP (2004) The puzzles of population cycles and outbreaks of small mammals solved? Bioscience 54: 1071-1079. Available online at: doi:10.1641/0006-3568(2004)054[1071:TPOPCA]2.0.CO;2

[75] Wang H, Nagy JD, Gilg O, Kuang Y (2009) The roles of predator maturation delay and functional response in determining the periodicity of predator-prey cycles. Math Biosci 211: 1-10. PubMed: 19563815. 10.1016/j.mbs.2009.06.004 19563815

[76] Scott S, Duncan CJ (1998) Human Demography and Disease. Cambridge: Cambridge University Press. 354 p.

[77] Kaner S (1996) Facilitator’s Guide to Participatory Decision-Making. Gabriola Island, BC, Canada: New Society Publishers . 255 p

[78] Wondolleck JM, Yaffee SL (2000) Making collaboration work: lessons from innovation and natural resource management. Washington, DC: Island Press. 277 p.

[79] Van den Belt M (2004) Mediated modeling: a system dynamics approach to environmental consensus building. Washington, DC: Island Press. 339 p.

